# Type material comparison of possible cryptic species of the genus *Electrogena* (Ephemeroptera, Heptageniidae) in Central Europe

**DOI:** 10.3897/zookeys.845.30095

**Published:** 2019-05-15

**Authors:** Marek Polášek, Jan Šupina, Roman J. Godunko

**Affiliations:** 1 Masaryk University, Department of Botany and Zoology, Kotlářská 2, CZ-61137 Brno, Czech Republic Masaryk University Brno Czech Republic; 2 Biology Centre of the Czech Academy of Sciences, Institute of Entomology, Branišovská 31, CZ-37005 České Budějovice, Czech Republic Biology Centre of the Czech Academy of Sciences, Institute of Entomology České Budějovice Czech Republic; 3 State Museum of Natural History, National Academy of Sciences of Ukraine, Teatralna 18, UA–79008 Lviv, Ukraine National Academy of Sciences of Ukraine Lviv Ukraine

**Keywords:** *
Electrogena samalorum
*, *
Electrogena ujhelyii
*, *
Electrogena rivuscellana
*, species inquirenda, synonymy, type series

## Abstract

The genus *Electrogena* Zurwerra & Tomka, 1985 is a diverse mayfly group in the Western Palaearctic with a partially unclear taxonomy, even in well-examined areas such as Central Europe. Recently, one of the species belonging to this genus, *Electrogenaujhelyii* (Sowa, 1981), was identified as a complex of genetically and geographically separated species. Two other species, *Electrogenasamalorum* (Landa, 1982) and *Electrogenarivuscellana* Sartori & Landolt, 1991 were formerly stated as junior synonyms of the earlier species. The fact that the synonymy of *E.samalorum* and *E.ujhelyii* was stated without comparison of any larval or adult material and both species reportedly have different altitude preferences makes the taxonomical position of *E.samalorum* (and possibly *E.rivuscellana*) questionable. Among others, a comparison of type series is one of the first methods that should be used to clarify the taxonomical position of closely related taxa.

The present study aims to comparatively examine the type material and topotypes of *E.ujhelyii* and its presumed junior synonym *E.samalorum* for the first time in detail. Additionally, some notes on the status of the geographically extralimital *E.rivuscellana* are discussed briefly. We noted a significant similarity of all studied material from both the larval and imaginal stages, and suggest considering both junior synonyms (*E.samalorum* and *E.rivuscellana*) as *species inquirendae*.

## Introduction

Despite a long history of research on European mayfly taxonomy and ecology, there are still several groups of taxa with unresolved and unclear taxonomy. One of those groups is the genus *Electrogena* Zurwerra & Tomka, 1985. The first described species of this genus was *Electrogenalateralis* (Curtis, 1834), and the last described European species of this genus thus far is *Electrogenabrulini* Wagner, 2017. The latter species was delimited from the closely related species *Electrogenagridellii* (Grandi, 1953) using mitochondrial gene sequence analyses. This illustrates the possible existence of closely related undistinguished species within this genus even in a well-examined area. In Central Europe, we identified a similar situation with two species recently considered to be synonyms, *Electrogenaujhelyii* (Sowa, 1981) and *Electrogenasamalorum* (Landa, 1982) as a junior synonym of the former species (Zurwerra and Tomka 1986).

The species *Electrogenaujhelyii* was reported for the first time as “*Ecdyonurussubalpinus* Klp.” by Sándor Újhelyi based on larval material collected in August 1957 and 1958 (Újhelyi 1966: 205) from Aszófö, a small tributary stream that empties into Lake Balaton. Later (in August 1975 and 1976), S. Újhelyi collected additional larval and imaginal material, which was passed to Ryszard Sowa (Kraków, Poland) for a study. The species *E.ujhelyii* was described based on that material and was originally attributed by Sowa (1981: 375) to the *lateralis* species group of the genus *Ecdyonurus* Eaton, 1868.

Landa (1969: 193) indicated *E.samalorum* for the first time in Czechoslovakia in a monograph as *Electrogenalateralis* (Curtis, 1834) [orig. *Heptagenialateralis*]. Vladimír Landa collected larvae in 1963 in the Poprad River in the town Podolínec, Slovakia. Later, V. Landa and Tomáš Soldán collected other material for *E.samalorum* in the Czech Republic and Slovakia (including type material from Podhorodský Stream in the village of Podhoroď, Slovakia). Finally, based on the material listed above *E.samalorum* was described as a new species for former Czechoslovakia (Landa and Soldán 1982).

A few years later, Zurwerra and Tomka (1986) established the synonymy of *E.ujhelyii* and *E.samalorum* with reference to personal communications with Dietrich Braasch (Potsdam, Germany) without any other details or comments. During our personal contact (RJG, October 2008) with D. Braasch, he reported that the conclusion on the synonymy of *E.ujhelyii* and *E.samalorum* was made by him based on an analysis of his own larval collection of *E.ujhelyii* from Germany in 1982 (the territory of the former German Democratic Republic), which in his opinion, corresponded to the description of both species at once (see also Braasch and Jacob 1984: 81–82). Later, Kluge (2004) and Bauernfeind and Soldán (2012) supported the synonymy. However, this was also determined without any details or argument. Nevertheless, in several other works (e.g. Landa and Soldán 1989; Deván 1989; Kłonowska-Olejnik 2003), *E.samalorum* has been indicated as a separate species, although its larvae (e.g. in Deván 1993) were compared with larvae of the species *E.lateralis*, but not of *E.ujhelyii.* In addition to the absence of a detailed material comparison, there is another detail that brings into question the synonymy of both species. Landa and Soldán (1982) stated that *E.samalorum* lives only in highlands, while Sowa (1981) reported lowland occurrences of *E.ujhelyii* (in the vicinity of Lake Balaton, approx. 150 m a.s.l.).

In addition to the synonymy of the two species discussed above, Belfiore and Desio (1995) proposed a synonymy between *E.ujhelyii* and *E.rivuscellana* Sartori & Landolt, 1991 based on a comparison of larval material from Austria, Italy (*E.ujhelyii*) and Switzerland (one paratype of *E.rivuscellana*). After analysing the set of 16 numerical characters, the authors noticed a considerable overlap of the main diagnostic qualitative and quantitative characters in mouthparts, gills, legs and femoral setation, most of which were not considered in the original description of *E.rivuscellana* (Landolt et al., 1991).

Most recent results on the genetic diversity of Central European representatives of the genus *Electrogena* show that specimens identified as *E.ujhelyii* belong to a complex of two morphologically indistinguishable but geographically separated species (Polášek et al. 2018). Moreover, the distribution areas of both cryptic species indicate that one of those indistinguishable species might belong to the former *E.samalorum*, while the other species can be assigned to *E.ujhelyii* sensu stricto due to the availability of collected topotypic material. The material from the type locality of *E.samalorum* (Podhoroď, Slovakia) was unfortunately not available, the only species found in the adjacent region belong to *E.lateralis*.

In this work, a revision and comparison of type material for both problematic Central European species was conducted for the first time, and we discuss possible larval and adult characters to distinguish between the species. Furthermore, some notes on the status of the extralimital *E.rivuscellana* are discussed briefly.

## Materials and methods

### Type material

**Type series of *Electrogenaujhelyii* (Sowa, 1981)**

The original description of the species *E.ujhelyii* is based on a male imago (holotype), four other male imagines, eight female imagines and seven mature larvae (all paratypes). All of the type material originated from the environs of the village of Tihany (Hungary) on the Aszófö stream and was first kept at the Department of Hydrobiology (Jagiellonian University, Poland) (Sowa 1981). In 1997, R. Sowa’s collection was transferred to the Musée Cantonal de Zoologie Lausanne (Switzerland). Finally, this material was provided at our disposal on a long-term loan in April 2012, and is provisionally housed at Masaryk University, Brno, Czech Republic.

At present, the available type material on *E.ujhelyii* includes only five specimens preserved in alcohol.

**Figure 1.**
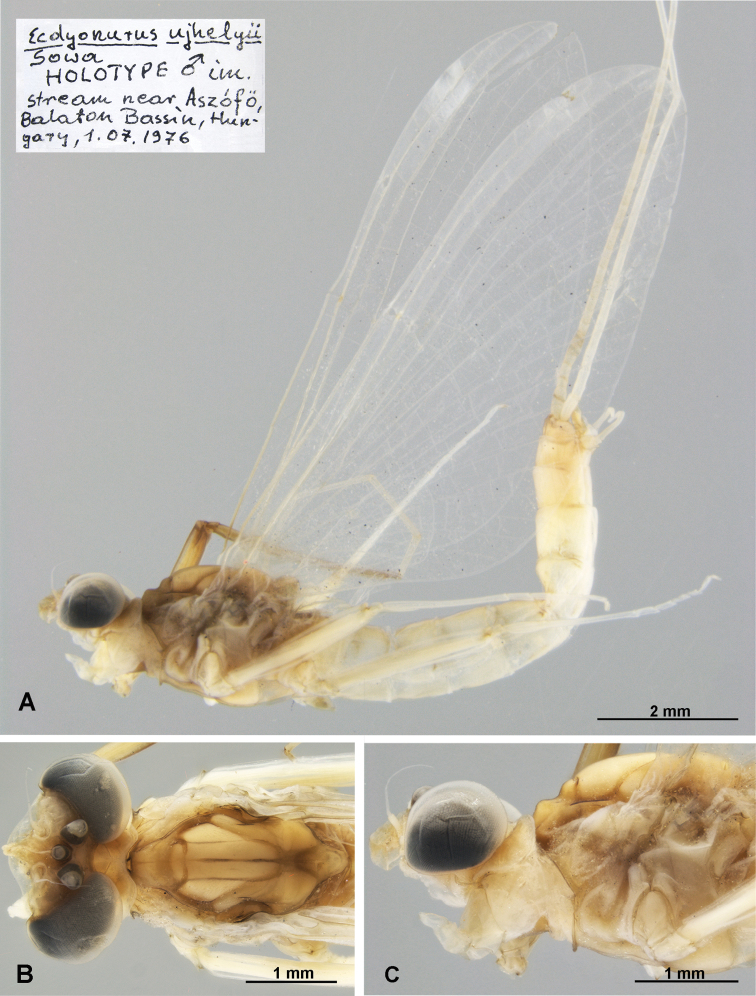
*Electrogenaujhelyii* (Sowa, 1981), male imago, holotype: **A** body, left lateral view **B** head and thorax, dorsal view **C** head and thorax, left lateral view.

**Figure 2.**
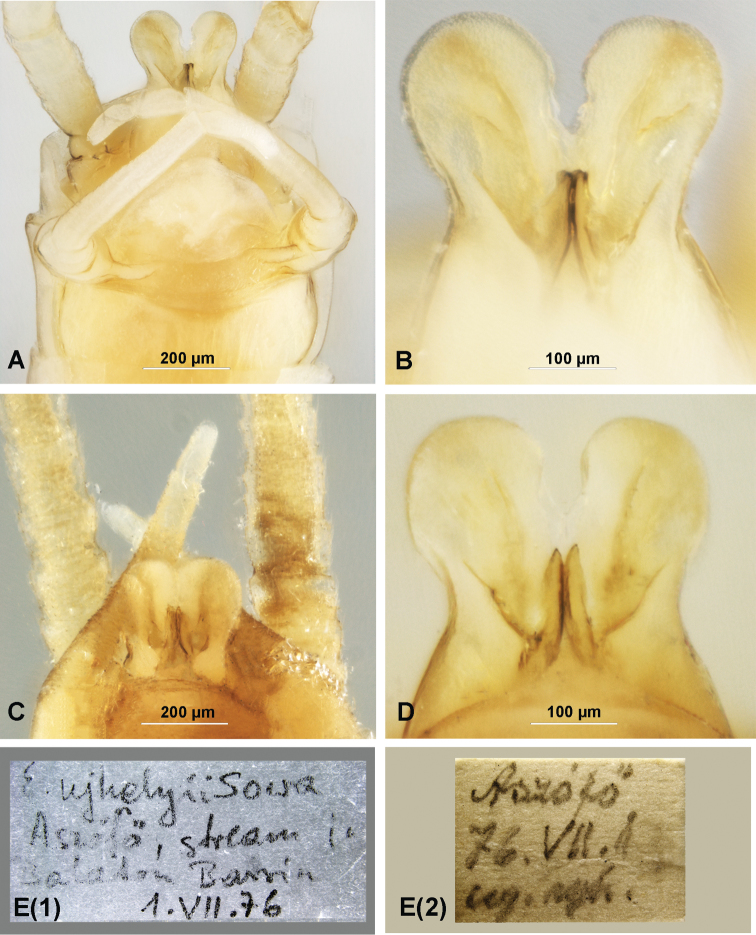
*Electrogenaujhelyii* (Sowa, 1981), male imagines, holotype (**A, B**); paratype I (**C, D**): **A, C** male terminalia, ventral view **B, D** penis lobes, ventral view **E(1), E(2)** original labels of paratype I.

(i) Holotype (Figs 1A–C, 2A, B), male imago, labelled as: “Ecdyonurusujhelyii Sowa Holotype ♂ im. stream near Aszófö, Balaton Bassin, Hungary, 1.07.1976”; well preserved specimen in a separate tube, missing the left foreleg and the right hindleg; S Újhelyi leg.

(ii) Paratype I (Fig. 2C, D), male imago, labelled as: “E.ujhelyii Sowa Aszófö, stream in Balaton Bassin 1.vii.76”; “Aszófö 76.VII.1 leg. Ujh.” (Fig. 2E(1), E(2)); well preserved specimen, missing the right foreleg and the left hindleg; in a tube together with paratype II and paratype III (see below); S. Újhelyi leg.

(iii) Paratype II, male imago, label is the same as for paratype I; the specimen was preserved during the moult, with subimaginal skin; S Újhelyi leg.

(iv) Paratype III, female imago with preserved eggs, label is the same as for paratype I; incomplete specimen, missing legs.

(v) Paratype IV (Fig. 4A, B), mature male larva, labelled as: “Hungary: Aszófö 10.8.1958 leg. Újhelyi”.

Holotype label was prepared by Małgorzata Kłonowska-Olejnik (Kraków, Poland) in the 1990s; labels of paratypes were written by R Sowa and S Újhelyi. Hitherto, any slides of other specimens of the type series or their parts were not preserved.

Besides the original type material collected by S. Újhelyi, we collected and analysed topotypic material from the Aszófőii-séd brook near Aszófő (GPS loc.: 46°56.0648'N, 17°49.8379'E) sampled on July 12, 2012. Altogether, we used seven mature larvae for subsequent analyses and comparisons.

**Type series of *Electrogenasamalorum* (Landa, 1982)**

The description of the species *E.samalorum* is based on imaginal and larval material, which was collected in the basins of the Elbe and Vistula Rivers. With the exception of the holotype (male imago reared from larva), female imago and two larvae, which have their localities, date of collection, and the name of collector clearly labelled, the collection data and other details for the other 13 larvae and three male imagines are indicated but not specified, which would be helpful for identification of the type material. Hence, when analysing the present volume of the type series of *E.samalorum*, we used the information from publications, original labels and the personal comments of T. Soldán. The type specimens, designated as *E.samalorum*, are preserved in alcohol and partly mounted on slides. Among the materials indicated as types of *E.samalorum*, we found two male imagines without genitalia in alcohol, labelled as “Brook–Valeč–Doupov North Bohemia–12.7.1956”. Marked locality and collection data correspond to that belonging to the paratype of female imago *E.samalorum* (see Landa and Soldán 1982: 34).

The identification of two males listed above has shown that one of them undoubtedly belongs to the type series *E.samalorum* based on the colouration of body, the colour pattern of lateral sides of abdominal terga II–VIII as described by Landa and Soldán (1982) and the structure of genitalia mounted on slide (see below). Another male imago probably belongs to the subgenus Helvetoraeticus of the genus *Ecdyonurus* according to the presence of the characteristic *L*-shape markings on the lateral sides of abdominal terga II–VIII, which is poorly visible due to the long-term preservation. It is possible that in this case we deal with the species *Ecdyonurussubalpinus* (Klapálek, 1907), mentioned by Landa (1969: 220) from Valeč village.

**Figure 3.**
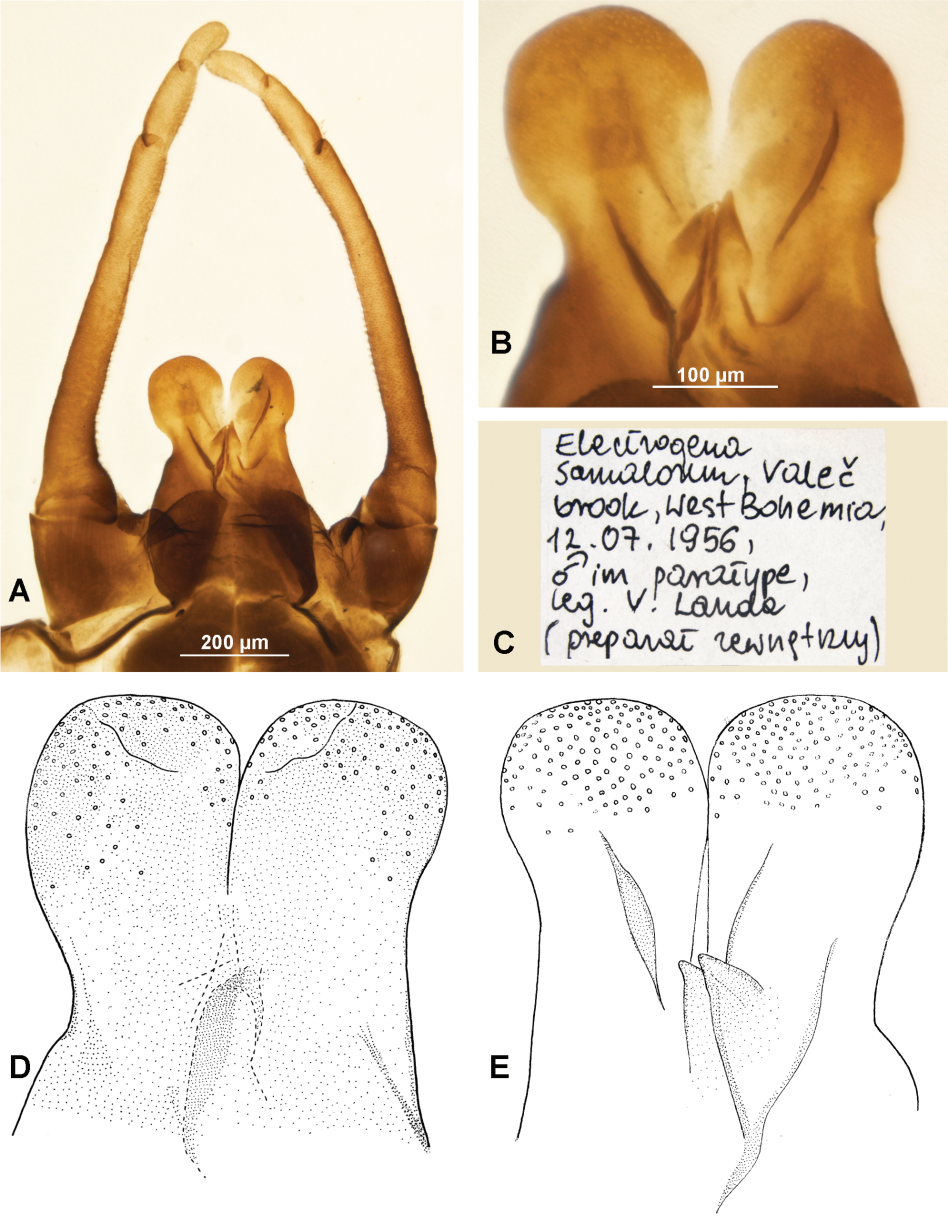
*Electrogenasamalorum* (Landa, 1982), male imago, paratype I (**A–C**), 12.vii.1956; male imago, not type (**D, E**), 29.vii.1961: **A** male terminalia, dorsal view **B** penis lobes, ventral view **C** label on microslide **D** penis lobes, dorsal view **E** penis lobes, ventral view.

Together with the material in alcohol in the collection of the Institute of Entomology (Biology Centre of the Czech Academy of Sciences), the slide with genitalia of two *E.samalorum* males, was found and mounted as a single slide and labelled by M Kłonowska-Olejnik during her stay at the Institute of Entomology, Biology Centre of the Czech Academy of Science in 2002. One of two genitalia belongs to the paratype of the male imago, discussed above, and is labelled as: “Electrogenasamalorum, Valeč brook, West Bohemia, 12.07.1956, ♂ im paratype, leg. V. Landa (preparat zewnętrzny) [external slide]” (Fig. 3A–C). The second male genitalia belong to the specimen of *E.samalorum*, and is labelled as: “E.samalorum Výrovka, Kostomlaty, 29.07.1961, ♂ im leg. V. Landa (preparat wewnętrzny) [internal slide]” (Fig. 3D, E). A complete specimen of this male has not been found and is probably lost; this specimen was not a part of type series.

**Alcohol material**:

(i) Paratype I (Fig. 3A, B), male imago, labelled as: “Brook–Valeč–Doupov North Bohemia–12.7.1956”; damaged specimen without forelegs, middle right leg, and hind left leg; genitalia mounted on slide; V Landa leg.

(ii) Paratype II, mature male larva; in same tube subimaginal skin also (probably belongs to the holotype male imago reared from larva); both labelled as: “Podhorod – paratypes”; significantly damaged larva; complete subimaginal skin; T Soldán leg.

(iii) Paratype III and paratype IV, male and female larvae, labelled as: “pot. u Lendak 25.7.61”; significantly damaged specimens; V Landa leg.

(iv) Paratype V and paratype VI, mature male larva and male larva, labelled as: “Heptagenia sp 1”, “Poprad / Podolince 24.6.1963”; significantly damaged larvae; V Landa leg.

(v) Paratype VII, mature female larva, labelled as: “Javorinka – Podspády / 26.8.63”; damaged larva; V Landa leg.

(vi) Paratype VIII, mature male larva, labelled as: “Potok u Maková / 18.6.63”; well preserved larva; V Landa leg.

(vii) Paratype IX, mature male larva, labelled as: “Pot. Lopušna / 18.6.63”; damaged larva; V Landa leg.

(viii) Paratype X, female larva, labelled as: “Pot. nad Brusnicou 27.7.61”; well preserved larva; V Landa leg.

(ix) Paratype XI and paratype XII, mature male and female larvae, labelled as: “Potok u Kastánie / 18.6.63”; damaged larvae; V Landa leg.

**Figure 4.**
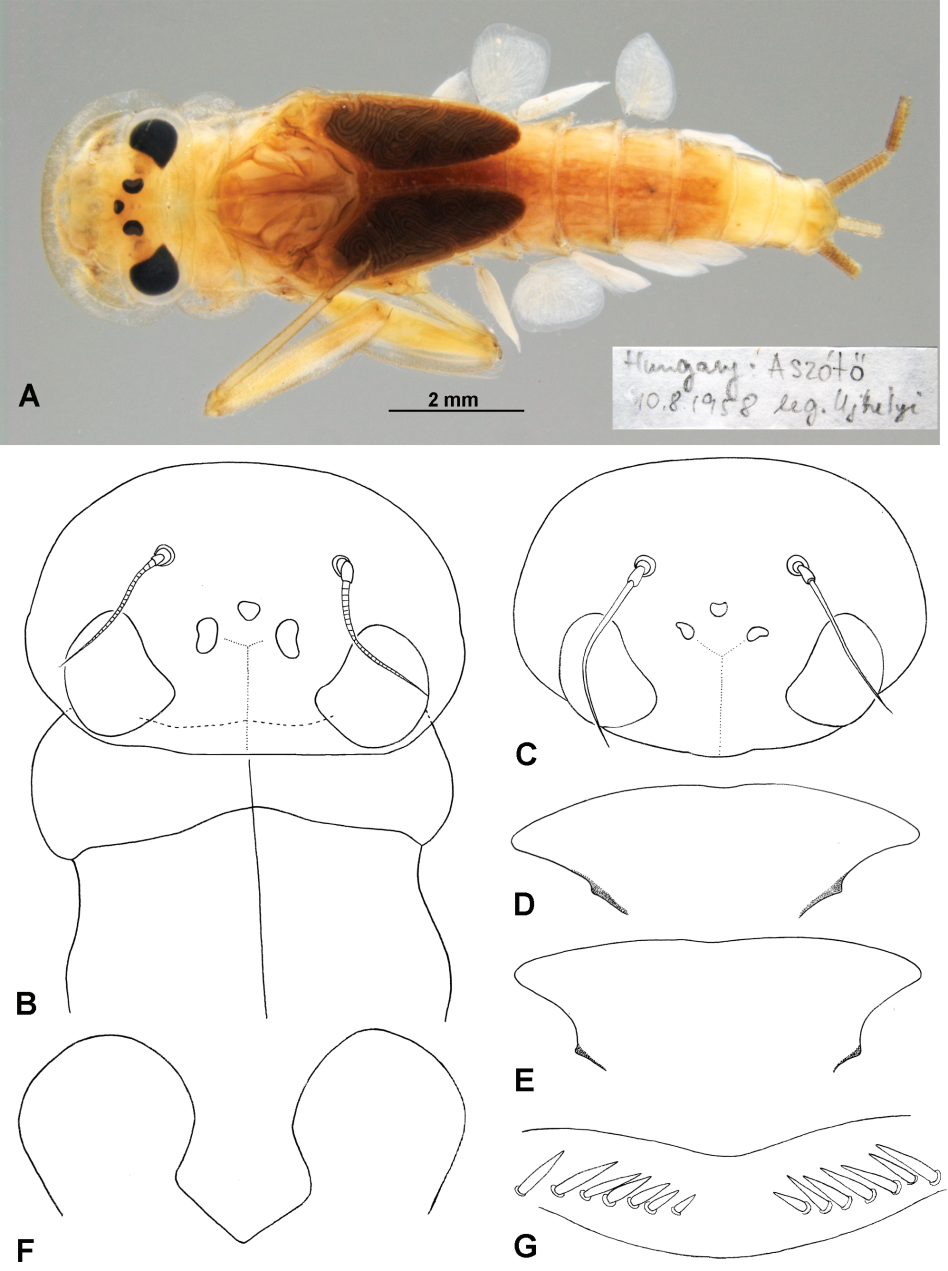
*Electrogenaujhelyii* (Sowa, 1981), mature male larva, paratype IV (**A, B**); *Electrogenasamalorum* (Landa, 1982), mature female (**C, E, G**) and mature male (**D, F**) larvae, paratypes XII and XIII: **A** body, dorsal view, 10.viii.1958 **B** head and pronotum, dorsal view, 10.viii.1958 **C** head, dorsal view, 16.vii.1975 **D** labrum, ventral view, 18.vi.1963 **E, G** labrum, ventral view, 16.vii.1975 **F** glossae, ventral view, 18.vi.1963.

**Figure 5.**
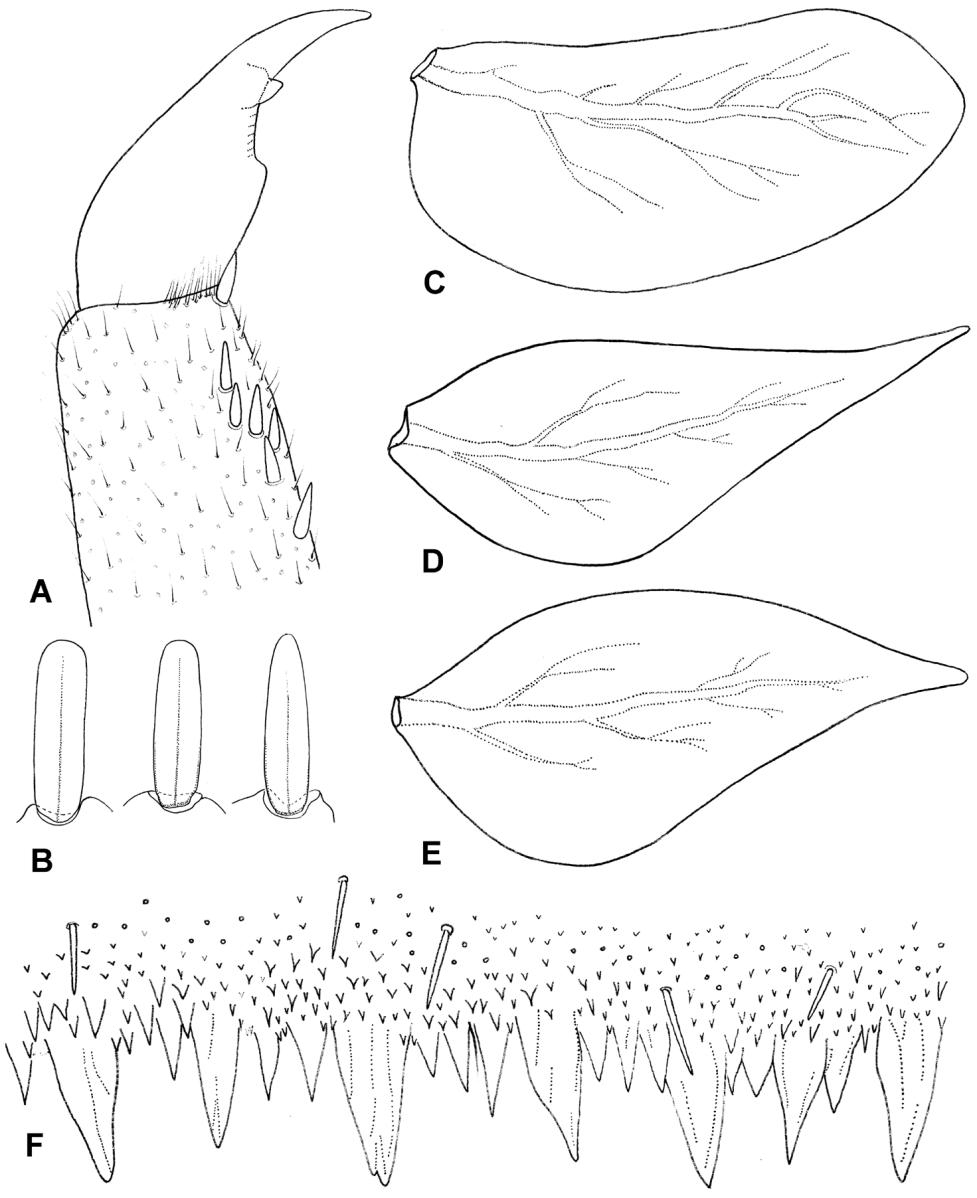
*Electrogenasamalorum* (Landa, 1982), mature male (**A, C, E**) and mature female (**D**) larvae, paratypes XII and XIII: **A** pretarsal claw, 18.vi.1963 **B** femoral setae, dorsal surface of hind femur (based on two mentioned paratypes) **C** gill I, 18.vi.1963 **D** gill 7, 16.vii.1975 **E** gill 7, 18.vi.1963 **F** tergite IV, posterior margin.

Slides (mounted based on paratypes XII and XIII):

(x) Paratype XII [mounted using paratype XII] (Figs 4C, F, 5A, B, D, F) and paratype XIII (Fig. 4E, G), female larvae, labelled as: “Electrogenasamalorum Podhorod, Podhorský brook, 16.07.1975, L ♀ paratype A [another slide labelled as “L ♀ paratype B”], leg. T Soldán”; slides mounted with Liquide de Faure and labelled by M Kłonowska-Olejnik.

(xi) Paratype XIII (Figs 4D, 5C, E), female larva, labelled as: “Electrogenasamalorum, Lopušna brook, 18.06.1963, L ♀ leg. V Landa”; idem.

(xii) male genitalia [paratype I (Fig. 3A, B); for details see above]; male genitalia [imago, not of type (Fig. 3D, E); for details see above]; idem.

In addition to the *E.samalorum* holotype and 19 paratypes indicated in the original description, hitherto, 13 paratypes have been preserved, of which three larval paratypes originated from the type locality. The holotype of the species has not been found, and is probably lost.

### Material processing

The line drawings of the morphological structures of the specimens belonging to the type series of Central European species were made using a Zeiss Axoiplan and Olympus BX41 microscopes, both equipped with a drawing attachment (camera lucida). The material was observed with a Leica M205 C binocular stereomicroscope; the eggs structure was investigated using phase-contrast microscope Di-Li 2026-P with 16 MP digital camera. Photographs of types were taken using a Leica Z16 APO microscope and processed with the Leica Application Suite™ Version 3.1.8 to obtain combined photographs with a suitable depth of field at the Staatliches Museum für Naturkunde Stuttgart. Photographs were subsequently enhanced with Adobe Photoshop™ CS3.

The numerical morphological characters defined by Carlo Belfiore for *Electrogena* species identification (Belfiore 1996, 1997) have been measured and minimum, maximum and mean value of each character was used for both species comparison. The preservation of available material did not allow to measure all defined characters; the characters based on number of fine setae or fragile body parts were thus omitted. The following characters were measured:

**N_CBS** number of comb-shaped bristles on fore margin of galea-lacinia;

**N_CLW** number of teeth on pretarsal claws;

**N_BVF** number of bristles on ventral side of femora near hind margin;

**R_LBR** total width of labrum divided by mean width of lateral lobes of labrum;

**R_GLA** relative distance between glossae (outer distance divided by inner distance between glossae);

**R_GLB** relative width of glossae (outer distance divided by width of glossae);

**R_1GI** relative width of 1^st^ gill plate;

**R_4GI** relative width of 4^th^ gill plate;

**R_7GI** relative width of 7^th^ gill plate.

For detailed description of the characters see Belfiore (1996, 1997) and Polášek et al. (2018).

## Results

### Male imagines

Figs 1–3

*Body colouration.* The natural colour pattern is poorly preserved, especially in the paratype of the male imago of *E.samalorum*. Most changes were caused by long-term storage in alcohols of different concentrations and qualities. In the holotype and paratypes of *E.ujhelyii*, the general colour of the body is pale, dirty yellow, yellowish-brown to brown. The body colour of the preserved paratype of *E.samalorum* is distinctly darker than in all of the *E.ujhelyii* adult type specimens, with a brown to dark brown head and thorax and brown abdominal segments. Such a difference in coloration may be the result of inter-population variability. Besides, the lack of new topotype material on adult specimens of both species at our disposal makes it impossible to define the limit between inter- and intraspecific variability in colour pattern.

*Head.* In *E.ujhelyii* the head is light brown without conspicuous maculation; compound eyes widely separated, the sclerite joining the compound eyes weakly rounded, forming a wide U (Fig. 1B) (the same for *E.samalorum* based on the study of type material); compound eyes bluish-grey to whitish-grey apically, and blackish towards the base, without distinct rings laterally (Fig. 1A, C) (in *E.samalorum*: bicoloured laterally, with light dirty brown upper 2/3, and blackish grey lower 1/3 of eyes height [*eyes whitish gray, dorsal surface darker without rings*: original description of *E.samalorum*]).

*Thorax*. In *E.ujhelyii* the thorax is slightly darker than other body parts, with distinct contrast maculation dorsally and laterally (Fig. 1A–C) [“*with slight violet pigmentation*”: original description of *E.ujhelyii*]: pronotum with a pair of triangular light brown spots laterally; mesonotum with brown median longitudinal, mesonotal and lateroparapsidal sutures; anterior part of the anteronotal protuberance of the mesonotum, area between the antelateroparapsidal suture and the sublateroscutum, and scutoscutellar impression (area between posterior scutal protuberances) with brown markings; metanotum uniformly light dirty brown (all terminology based on Kluge 2004). Lateral and ventral sides of thorax light dirty brown, with brown anterior paracoxal suture. In *E.samalorum* the orientation of markings is the same as in *E.ujhelyii* holotype and paratypes, but the general coloration of thoracic structures is distinctly darker, with marked brown sutures and respective maculation [“*thorax dark brown*”: Landa and Soldán (1982)].

*Legs.* The forelegs of *E.ujhelyii* distinctly darker than the middle and hind legs (Fig. 1A); forefemur and foretibia intensely brown, markedly dark brown apically (the same in original description of *E.samalorum*); middle and hind legs uniformly whitish-yellow, transversal band on middle and hind femora not preserved (diffuse unclear macula in paratype of *E.samalorum*; the same in original description of *E.ujhelyii*).

*Wings* of *E.ujhelyii* translucent, pterostigmatic area dirty milky or yellowish coloured; longitudinal and transversal veins yellowish (the same in male paratype of *E.samalorum*). The characters of the veins’ colouration are different in both the original descriptions and preserved types, probably due to long-term storage in alcohol.

*Abdominal segments. E.ujhelyii*: light yellow to dirty yellowish-brown, segments (I) II–VI translucent or not transparent; traces of preserved dirty violet maculation relatively visible on abdominal terga II–VIII (poorly visible in holotype, more distinct in both paratypes); the pattern of abdominal terga similar to those described and figured by Sowa (1981: 376–377, fig. 1) and Bauernfeind and Soldán (2012: 299), but differs from the colour pattern figured by Haybach (2005: 42–43, fig. 5), with a dirty violet spot basolaterally near the anterior and posterior margins of the segments (the spot near the posterior margin of segments poorly visible). The sterna are uniformly yellowish brown; the ganglionic chain is not visible.

In the male paratype of *E.samalorum* the pattern of the abdominal terga poorly visible, but nevertheless corresponds with the original description (Landa and Soldán 1982: 32): a pair of elongated brown strokes (occasionally fused together on terga II, IV, and VI) near anterolateral margin of segments.

*Genitalia*. *E.ujhelyii* (Fig. 2A–D): The base of the forceps with small asymmetrical apically rounded projections laterally, broadly convex in the middle; the forceps yellowish-brown to brown, and paler distally (Fig. 2A, C); the penis lobes stout and divergent apically with relatively wide interspace, U or V shaped; the lobes widely rounded apically or slightly truncate along the outer margin [the similar shape of lobes is depicted by Sowa 1981: 376, fig. 2; Bauernfeind and Soldán 2012: 594, fig. 188], the lobes moderately expanded laterally; both ventral folds of the lobes well developed (Fig. 2B, D); a few small pointed setae occur basodorsally (the same as in the original description of *E.ujhelyii*). The titillators relatively broad, slightly asymmetrical, bluntly pointed at the tip, straight basally and strongly bent apically (ventral edge sinuous).

The structure of forceps base and penis lobes of the male paratype of *E.samalorum* completely corresponds with the original description (Fig. 3A, B, D, E; see also figs 2–4 in Landa and Soldán 1982: 33). The male genitalia of the specimen collected 29.vii.1961 generally with similar structure of penis lobes. However the lobes are closely touching, without deep interspace (Fig. 2D, E; the same shape of penis lobes is depicted for *E.ujhelyii* by Landolt et al. 1991: 467, fig. 8).

### Female imago

Only a single specimen of the female paratype of *E.ujhelyii* has been preserved hitherto. The preserved colour of the body is similar to those in male specimens. Compound eyes uniformly black, with thin brownish edging. The shape of subgenital and subanal plates corresponds to those described and figured by Sowa (1981: 377, fig. 3) and Haybach (2005: 42–43, fig. 2a, b). A single female paratype of *E.samalorum* has most probably been lost.

### Eggs

The structure of eggs is described and figured in details for *E.ujhelyii* by Sowa (1981: 377), Haybach (2005: 42–43, fig. 8) and Bauernfeind and Soldán (2012: 300, 631, fig. 188), and for *E.samalorum* by Landa and Soldán (1982: 32, 37, pl. 1, figs 1–4). We have studied egg chorion structure in the species *E.ujhelyii*, based on the material prepared from a single preserved female paratype. The result of our observations completely corresponds with the information published previously for both species.

### Mature larva

Figs 4, 5

*Body colouration.* Like in adult specimens of the type series of *E.ujhelyii* and *E.samalorum*, the cuticular colouration of the studied paratypes of the larvae was also poorly preserved. Nevertheless, some aspects of colour pattern, which are important for identification and comparison of the taxa, are visible enough. The general colouration of the body in larvae of *E.ujhelyii* and *E.samalorum* is yellowish-brown to brown; a variable colour pattern is more or less preserved on the thorax, legs and abdomen.

*Head* of *E.ujhelyii* (paratype and topotypes) with broadly rounded anterior margin, the broadest part near to anterior end of eyes (Figs 4A, B); in *E.samalorum* the head capsule of similar shape, or the posterior margin occasionally truncated (Fig. 4C). The head colour of *E.ujhelyii* uniformly light brown to brown, without visible spots (the same in Landa and Soldán 1982; Bauernfeind and Soldán 2012), occasionally two small yellowish spots between the eyes and the lateral ocelli. In larval paratypes of *E.samalorum* the same head colour is observed; occasionally additional yellow spot between the base of the antennae and two smaller spots on both sides of the central ocellus. The labrum in *E.ujhelyii* and *E.samalorum* are shaped differently, slightly bent backwards or rather straight (Fig. 4D, E; Sowa 1981, fig. 4; Landa and Soldán 1982, fig. 8); a row of a relatively stout bluntly pointed setae available on the ventral side of the labrum (Fig. 4G).

*Thoracic* colour pattern is variable in both species, but generally corresponds to those in the original descriptions. Generally, the pronotum with two elongated transversal whitish spots near the anterior margin; the lateral sides of the pronotum regularly rounded, slightly stretched laterally with two yellowish strokes along the outer margin (Fig. 4B). The mesonotum with several elongated whitish-yellow markings of variable shape, and two diffuse spots near the wing pad bases in *E.ujhelyii* and *E.samalorum*.

*Legs*. The coxae of both species with distinct brown marking around the segment; the femora yellowish-brown with a pair of transversal zigzag-like light brown spots proximally and distally; the tip of the femora light brown; tibiae contrasted, with ¾ of the distal part light brown and ¼ of the proximal part yellow; tarsi light brown and slightly darker at the tip; pretarsal claw brown with 2–4 teeth (occasionally with one strong tooth, Fig. 5A). The characters of leg setation (including dorsal femoral setation) correspond with those in contributions published by Sowa (1981), Landa and Soldán (1982), Haybach and Belfiore (2003) and Bauernfeind and Soldán (2012).

*Abdominal segments* of both species with two pairs of diffuse yellow to light brown spots on a brown background on terga (I) II–VII (a pair of smaller spots close to the anterior margin of the segment centrally; a second pair of spots near the posterior margin of the segment laterally); tergite VIII occasionally with a large diffuse light spot centrally; tergite IX with a diffuse U-shaped light spot centrally; tergum X with elongated yellow spots centrally and smaller spots laterally (generally the same colour pattern was described for *E.samalorum* by Landa and Soldán 1982). Some differences occur between the observed shapes of the 1^st^ and 7^th^ gill plates in paratypes and topotypes of *E.ujhelyii* and *E.samalorum*, and drawings in the original descriptions are noted (see Fig. 5C, D, E). Nevertheless, in all materials studied, the 7^th^ gill abruptly tapers in its distal third. Additionally, Landa and Soldán (1982: fig. 12) depicted a row of broad nearly triangular or rectangular jagged teeth apically along the posterior margin of the abdominal terga. All observed specimens can be characterised by the presence of a mainly regular row of stout triangular teeth, alternating with a group of 1–3 smaller teeth (Fig. 5F).

**Table 1.** Minimum, mean and maximum values of some numerical characters for *E.ujhelyii* and *E.samalorum*, based on the larval type and topotype material.

**Numerical characters**	*** E. samalorum ***			*** E. ujhelyii ***		
	**min**	**mean**	**max**	**min**	**mean**	**max**
N_CBS	13	15.14	17	13	15.50	20
N_CLW	2	2,43	3	2	2,83	4
N_BVF	19	23.6	27	13	32,61	49
R_LBR	4.31	4.95	5.27	5.31	5.82	5.86
R_GLA	3.33	3.46	3.50	3.16	4.21	4.26
R_GLB	2.78	2.79	2.86	2.00	2.93	3.11
R_1Gl	2.18	2.29	2.33	1.97	2.21	2.47
R_4Gl	1.09	1.17	1.30	1.18	1.26	1.39
R_7Gl	2.23	2.48	2.76	2.15	2.35	2.54

### Numerical characters

The comparison of minimum, mean and maximum of selected numerical characters is presented in Tab. 1. We did not find any reliable character for delimitation of both studied species using the defined set of numerical characters. The range of all characters strongly overlapped, usually with values of those for *E.samalorum* within the range of *E.ujhelyii*. The only character with apparent difference between both species was R_LBR (total width of labrum divided by mean width of lateral lobes of labrum). Nevertheless the preservation of type material (namely of *E.samalorum*) and thus the number of measurements of this character did not allow the statistical evaluation.

## Discussion

In our comparison of the *E.ujhelyii* and *E.samalorum* type material and our attempt to reliably decide the taxonomical position of both species we encountered many obstacles. In contrast to the well-preserved type material and available topotypic material of *E.ujhelyii*, there are only sparse specimens of type material of *E.samalorum*. Notably, the holotype of *E.samalorum* is missing and part of remaining material is heavily damaged. In addition, we were unsuccessful at collecting topotypic material in eastern Slovakia. Using a unified set of numerical larval characters suggested by Belfiore (1996, 1997) has turned out to be impractical for the separation of these taxa. The preservation and number of type materials did not allow us to compare both taxa with sufficient credibility. The only character which differed between both species was R_LBR. However, this character is highly variable, the value of this character for *E.ujhelyii* varies from 3.7 to 5.9 (see Supplement in Polášek et al. 2018). For this reason, we considered this difference as a random result of insufficient number of measurements.

Thus, we have failed to find any substantial differences between the type material of *E.ujhelyii* and *E.samalorum* for all stages investigated. Only a few peculiarities in body coloration of *E.samalorum* in comparison with *E.ujhelyii* have been noted. In males (**i**) the body is markedly darker; (**ii**) the compound eyes have a relatively distinct border between the paler top and the darker base; (**iii**) the abdominal terga have brown strokes near the anterolateral margin of the segments only. In larva (**iv**) the head occasionally has two additional pairs of small spots on frons and vertex. However, we can assume that some of these differences may be related to the material storage mode during the past 35 years. As a consequence, the additional information for the delimitation of *E.ujhelyii* and *E.samalorum* based on the characters of colour pattern, which was recently discussed and successfully used by Wagner et al. (2017), was inaccessible to us. Moreover, these small differences between *E.ujhelyii* and *E.samalorum* might be a result of intraspecific variability of *E.ujhelyii*, as was mentioned by Sowa (1981) in the original description. On the other hand, recent research on the genetic diversity of Central European *Electrogena* species indicates that specimens identified as *E.ujhelyii* actually belong to two geographically distinct species (Polášek et al. 2018).

The status of *E.rivuscellana*, described for the first time from Switzerland (Landolt et al. 1991) can also be considered as problematic. The synonymy was stated by Belfiore and Desio (1995) by examination of only one larval skin, probably the paratype using Carlo Belfiores’ system of numerical characters. However, limited usability of this system for closely related species delimitation has been proven in Polášek et al. (2018). Moreover, there are some differences in published larval and adult morphological characters which could make the recent synonymy questionable. In contrast to the types of *E.ujhelyii* and *E.samalorum*, the genitalia of *E.rivuscellana* is described as follows: (**i**) the penis lobes are visibly stretched laterally, with a relatively obtuse outer margin and posterolaterally the constriction of lobes is marked by a notch (giving the impression of small hump under the lobes); (**ii**) the forceps base has lateral protuberances which rise above the central convexity; and (**iii**) the titillators are comma-shaped, short and broadly rounded from both sides (see Landolt et al. 1991: figs 4, 5, 18–20, 22; Malzacher 1996: 84, figs 2, 5a, c, d). The larvae differ in the following details: different shape of the first gill plate (widest in the distal part in *E.rivuscellana* and in the proximal part in *E.ujhelyii*, see Landolt et al. 1991: fig 14; Sowa 1981: fig 7), different shape of the 4^th^ gill plate (more quadrancular in *E.rivuscellana*, see Landolt et al. 1991: fig 15; Sowa 1981: fig 8) and slightly different body coloration in *E.ujhelyii* (violet spots on abdominal segments, violet ganglia, Sowa 1981). Despite two recent studies on genetic diversity of *Electrogena* species including *E.ujhelyii* (Wagner et al. 2017; Yanai et al. 2017), no data about Swiss populations of *E.ujhelyii* (and therefore possibly *E.rivuscellana*) have been published thus far except one analysed specimen of *E.ujhelyii* s. str. in Polášek et al. 2018, and could therefore be a possible case of cryptic diversity too.

## Conclusions

We noted a remarkable similarity of all studied material from both larval and imaginal stages of *E.ujhelyii* and *E.samalorum*. However, due to the considerable damage of studied material and some noted differences between *E.ujhelyii* and *E.samalorum* type material, we cannot presently confirm their synonymy which was previously established without comparison of any material. Moreover, the recently revealed cryptic intrageneric diversity and existence of (at least) two morphologically nearly identical species with geographical distribution overlapping with type localities of *E.ujhelyii* and *E.samalorum* makes their synonymy even more doubtful. For similar reasons, the taxonomical position (suggested synonymy) of *E.rivuscellana* can be also considered as problematic. We found some significant differences in *E.ujhelyii* and *E.rivuscellana* larval and adult morphology based partially on comparison of drawings and descriptions in available literature. Additionally, the synonymy has been established using Carlo Belfiores’ system of numerical characters which using must be considered carefully due to the limitations revealed in our last publication (Polášek et al. 2018). Given these uncertainties, it would be imprudent to assume synonymy of these species and we suggest that both species (*E.samalorum* and *E.rivuscellana*) be considered not as junior synonyms, but as *species inquirendae*.
